# Quantifying the Growth of Glioblastoma Tumors Using Multimodal MRI Brain Images

**DOI:** 10.3390/cancers15143614

**Published:** 2023-07-14

**Authors:** Anisha Das, Shengxian Ding, Rongjie Liu, Chao Huang

**Affiliations:** Department of Statistics, Florida State University, Tallahassee, FL 32306, USA; ad20fx@fsu.edu (A.D.); nding2@fsu.edu (S.D.); rliu3@fsu.edu (R.L.)

**Keywords:** glioblastoma (GBM), malignant cells, radiomics, proliferation, Bayesian technique, posterior mean

## Abstract

**Simple Summary:**

Prediction of volume expected to be attained by a tumor of fourth grade malignancy becomes difficult when problem is subject to changes in time or when there exists heterogeneity among oncogenes for different subjects. The attempt here was to develop a time independent model which will only depend on some other radiomic properties of the tumor, also accommodating for the heterogeneity feature. Our model gives highly accurate results when we subject the initial volume of the tumor and eventually discover that the probability of no tumor cells remaining undetected is sufficiently high. Our model results are for glioblastoma tumor, but it can be applied on any other tumor volume prediction problem, and especially can be reliably adopted for tumors of high malignancy level.

**Abstract:**

Predicting the eventual volume of tumor cells, that might proliferate from a given tumor, can help in cancer early detection and medical procedure planning to prevent their migration to other organs. In this work, a new statistical framework is proposed using Bayesian techniques for detecting the eventual volume of cells expected to proliferate from a glioblastoma (GBM) tumor. Specifically, the tumor region was first extracted using a parallel image segmentation algorithm. Once the tumor region was determined, we were interested in the number of cells that could proliferate from this tumor until its survival time. For this, we constructed the posterior distribution of the tumor cell numbers based on the proposed likelihood function and a certain prior volume. Furthermore, we extended the detection model and conducted a Bayesian regression analysis by incorporating radiomic features to discover those non-tumor cells that remained undetected. The main focus of the study was to develop a time-independent prediction model that could reliably predict the ultimate volume a malignant tumor of the fourth-grade severity could attain and which could also determine if the incorporation of the radiomic properties of the tumor enhanced the chances of no malignant cells remaining undetected.

## 1. Introduction

In recent years, liquid biopsy techniques have emerged as a non-invasive method to screen for cancer and monitor its progression. This involves analyzing blood or cerebrospinal fluid (CSF) samples to detect cancer-specific biomarkers, such as circulating tumor DNA (ctDNA), that can provide insight into the genetic mutations present in a tumor. These techniques have shown promising results in detecting and monitoring brain cancer and are being explored as a potential alternative to invasive biopsy procedures [1,2,3,4,5]. Another promising avenue of research in brain cancer diagnosis and treatment is the use of imaging techniques, such as magnetic resonance imaging (MRI) and positron emission tomography (PET) [2]. These techniques allow for the visualization of the tumor and its surrounding vasculature, thereby providing valuable information on the size, location, and bloodflow to the tumor, which are of great importance in treatment planning and prognosis. For example, certain PET tracers can be used to visualize the angiogenic activity of a tumor and predict its response to anti-angiogenic therapies [6,7,8]. Thus, overall, the field of brain cancer diagnosis and treatment has seen many advances in recent decades, which offers opportunities to better understand the biology of brain tumors and to predict their behavior [8,9].

The literature on the biology and treatment of glioblastoma multiforme (GBM), a central nervous system tumor of the fourth-grade histological astrocytic primary brain malignancy [10] as classified by the World Health Organization (WHO), highlights the need for more precise and accurate diagnostic methods to detect this type of brain tumor at its early stages [11] so that survival of the patient is possible upon surgical extraction of the tumor. However, although the available treatment options, including surgery, radiotherapy, and chemotherapy, have improved over time, their effectiveness and prognosis still depend on several factors, including the location and degree of malignancy of the tumor [12,13], the patient’s age, and the genetic profile of the tumor cells [14,15]. GBM happens to be a deadly tumor even to this date, and it takes a huge death toll every year in the United States alone [16,17,18,19]. Although various studies have attempted to predict the growth and progression of the brain tumor by incorporating different variables, such as the rate of proliferation and the migration of malignant cells, they cannot actually hold for this particular deadly tumor. For example, Rockne et al. (2010) used a biologically based linear–quadratic model to predict the response to magnetic resonance imaging (MRI) in individual patients, but the study did not account for the impact of treatment on the size of the tumor [20], which is crucial if surgical extraction needs to be adopted immediately once the cancer is detected. Stensjoen et al. (2015) proposed a Gompertzian growth model and a linear radial growth model that incorporated major variables to predict the growth of the tumor [21], but this happens to be time-dependent, which might not give the exact growth estimate expected up to its survival time. This looks like a potential risk, given the fatal nature and severity of the tumor. Yuan et al. (2016) compared the glial components of the surrounding tissues and found that the comparison of the bulk and adjacent astrocytes and microglia were the strongest predictors of survival, thus indicating the importance of the parenchymal components in predicting survival in GBM patients [17]. This study was a highlight regarding the ongoing efforts to improve our understanding of the biology and treatment of GBM, but still there is a dire need for more accurate diagnostic methods to detect this type of brain tumor at its early stages.

In dealing with a deadly tumor such as glioblastoma, radiomics analysis has proven to be a key tool for perceiving its analytic features [10], such as the prediction of clinical, proteomic (e.g., Ki-67 expression), genomic (e.g., IDH1 status) and transcriptomic parameters [22,23,24]. Furthermore, the utilization of T1, T1-Gd, and FLAIR image sequencing has led to recent developments in describing radiomic subtypes, each of which is an indicator of a unique phenotype supplement, such as MGMT methylation and EGFRvIII mutations [25]. A study conducted by Wang et al. (2016) showed that the combination of the diffusion tensor imaging (DTI) technique and the dynamic susceptibility contrast MRI technique could enhance precision in the treatment response and may foster the individualized treatment of patients with GBM [26]. Recent efforts have also shown that radiomics analysis is helpful in an efficient tumoral evaluation [25,27,28], along with the prediction of histo-pathological parameters, such as isocitrate dehydrogenase mutations [29,30,31,32] and ATRX mutations [33], among others. Radiomics also provides a scope to deal with the under-representations from contrasting clonal groups and to describe methodologies that might have significant contributions in intratumoral heterogeneity [34,35,36,37,38,39].

In this paper, the main goal of our research is an attempt to implement tumor segmentation and develop a radiomics model for the precise prediction of the growth of a glioblastoma up to its survival time. Earlier, there has been little to no attempt to develop a time-independent prediction model for a tumor as deadly as a fourth-grade malignant tumor, where surgical extraction is the only hope for the patient to survive. As a result, the detection mechanisms and the subsequent prognosis of such tumors are also not strong enough. The proposed approach aims to make use of the multimodal MRI images and demographic information of patients to predict the eventual volume of a glioblastoma multiforme. The eventual volume is calculated based on the number of tumor cells, including both newly born and migratory cells. The proposed model additionally takes into account some of the radiomic parameters of the tumor obtained through image segmentation, such as the morphology, spatial, and histology parameters, along with the intensity of the tumor images, as well as demographic factors such as age and gender of the patient. It should be noted that we only use information pertaining to the survival length of the tumors for each patient, and our model does not depend on time or change points. Thus, the final estimate is expected to be more precise and not subject to changes. The remaining parts of this paper are organized as follows. In Section 2, we provide details on extracting the tumor region using image segmentation techniques, along with the proposed Bayesian tumor cell growth model and the parameters used in the proposed model. In Section 3, we evaluate our model through both simulation studies and a real data example. All the analyses were performed using R software version 4.2.2. In Section 4, we discuss the potential implications of the proposed approach and in Section 5, we conclude and suggest areas for future research. The proposed approach could have important implications for improving the prognosis and lowering the risk for glioblastoma patients.

## 2. Materials and Methods

### 2.1. Data Information

The dataset considered in this study is a part of the TCGA-GBM collection that includes the preoperative baseline multimodal MRI scans provided by 102 patients from eight institutions all over the United States [40]. The available MRI modalities include T1-weighted precontrast (T1), T1-weighted post-contrast (T1-Gd), T2 and T2-FLAIR image intensities. For each patient detected with a GBM tumor, we have the volume of the tumor measured from different regions/angles, along with the volume of the whole tumor. The regions of interest (ROIs) in our study include ET (GD-Enhancing tumor), TC (tumor core) and WT (whole tumor). There are some tumor subregions which we also considered as ROIs. These include ED (edema) and NET (nonenhancing tumor). The survival length of the tumor (measured in days) is also available for each patient. We found that the maximum survival length of the tumors was approximately 7.6 years, while the minimum was zero. A brief descriptive analysis of the data in hand are summarized in Table 1, where all the radiomics features will be used later in a Bayesian regression analysis.

### 2.2. Preprocessing and Image Segmentation

**Figure 1:**
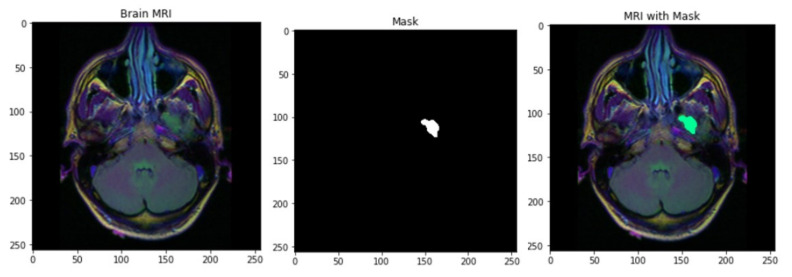
Tumor segmentation steps for one randomly selected subject, including the mMRI image (**left**), the tumor mask image (**middle**), and the highlighted tumor region in the mMRI image (**right**).

Similar to the radiomics analysis in Lu et al. (2018) [41], we followed a preprocessing and image acquisition step, as well as the identification of the radiomic features related to the molecular characteristics of the tumor [41,42]. Specifically, each of the preoperative multimodal MRI volumes were accommodated to the LPS (left posterior superior) coordinate system by making use of affine registration through the Oxford center for Functional MRI Linear Image Registration Tool and were resampled to a rate of one cubic millimeter of voxel resolution [40,43,44,45,46,47]. We used the Brain Extraction Tool in PyTorch and obtained the masked images individually. This enabled us to figure out the volumetric region occupied by the tumor before we moved on to predict its eventual volume until its survival time. The algorithm used behind this is simply a parallel segmentation of the mMRI images involving two steps that make use of *K*-means clustering. First of all, the location of the tumor is detected using the four types of image intensities. By making use of *K*-means clustering and morphological operation, we could incorporate the multiple image intensities corresponding to a single patient. This is followed by masking of the images for each subject. This method was earlier proposed by Dessai et al. (2012) [48]. Through the preprocessing and tumor segmentation, we extracted the tumor region for each subject. Figure 1 shows the tumor segmentation steps implemented for one randomly selected subject.

In addition, we considered several radiomic features of the extracted brain tumors, which were considered on the basis of manually revised labels of each tumor subregion, including intensity, morphologic [49,50,51,52], histogram-based [53], and textual parameters depending on wavelets [54], gray-level co-occurrence matrix (GLCM) [55], gray-level run-length matrix (GLRLM) [52,56,57,58,59], gray-level size-zone matrix (GLSZM) [56,57,58,60], and neighborhood gray-tone difference matrix (NGTDM) [61]. Furthermore, we also made use of some spatial information [62] and glioma diffusion properties from glioma growth models [63,64,65], which already have pre-existing predictive and prognostic values [53,66,67,68,69]. Information pertaining to all these attributes were extracted from denoised and unsmoothed images [70]. Our analysis does not involve studying the amount of biological significance of these radiomic features, but we tried to figure out the degree of dependence of them on the growth in volume of the tumor up to the given survival time of the tumor for the respective patients.

### 2.3. Quantification of the Tumor Growth Prediction

Some notations are introduced here. First, let $R^{\left(t\right)}$ be the set of voxels containing tumor cells at the *t*-th step of GBM tumor migration process, 0≤t≤T. We assume that (i) the baseline $R^{\left(0\right)}$ indicates the detected tumor region when the subject is diagnosed as a GBM patient; (ii) $R^{\left(T\right)}$ indicates the brain region containing existing and newly born tumor cells until the survival time of the tumor; and (iii) $R^{\left(t\right)}\subset R^{\left(s\right)}$ if 0≤t≤s≤T. In particular, the number of voxels in the incremental tumor region, i.e., $\Delta R^{\left(t\right)}≐R^{\left(t\right)}-R^{\left(t-1\right)}$, is always positive. Next, at the *t*-th tumor migration step, let $m_{t}$ be the number of (malignant and benign) cells proliferating in $\Delta R^{\left(t\right)}$, let $m_{t}^{*}$ be the number of corresponding malignant cells distinctly proliferating in $\Delta R^{\left(t\right)}$, let $M_{t}$ collectively be all the (malignant and benign) cells that proliferated until the *t*-th tumor migration step, i.e., $\sum_{s\leq t}m_{s}$ and $M_{t}^{*}$ are collectively all the corresponding malignant cells that proliferated until the *t*-th tumor migration step, i.e., $\sum_{s\leq t}m_{s}^{*}$. In addition, we denote that ν and $\nu^{*}$ are collectively all the (malignant and benign) cells and malignant cells, respectively, that can proliferate at the final tumor migration step.

In this paper, we propose a Bayesian analysis model to quantify the GBM tumor growth according to the volumes of regions of interest (ROIs) within the tumor area. First, given $\nu^{*}-M_{t-1}^{*}$ malignant tumor cells and $\left(\nu -\nu^{*}-M_{t-1}+M_{t-1}^{*}\right)$ benign ones undetected after the (t−1)-th tumor migration step, the joint probability mass function of the number of tumor cells and the number of malignant ones proliferating in $\Delta R^{\left(t\right)}$ can be represented as follows:

(1) $$p\left(m_{t},m_{t}^{*}|M_{t-1},M_{t-1}^{*},\nu ,\nu^{*}\right)=p\left(m_{t}^{*}|m_{t},\nu -M_{t-1},\nu^{*}-M_{t-1}^{*}\right)\times p\left(m_{t}|\nu -M_{t-1}\right),$$

where $p(m_{t}^{*}|m_{t},\nu -M_{t-1},\nu^{*}-M_{t-1}^{*})$ indicates the probability that exactly $m_{t}^{*}$ malignant tumor cells and $\left(m_{t}-m_{t}^{*}\right)$ benign ones are detected at the *t*-th step, which can be derived through the hypergeometric distribution as follows:

(2) p(mt*|mt,ν−Mt−1,ν*−Mt−1*)=ν*−Mt−1*mt*ν−ν*−Mt−1+Mt−1*mt−mt*ν−Mt−1mt,

and $p(m_{t}|\nu -M_{t-1})$ can be taken to be Poisson with the expectation νλ under the assumption that no recovery takes place until one cell has proliferated at the *t*-th step. Then, the joint distribution of ${\left\{m_{t},m_{t}^{*}\right\}}_{t=0}^{T}$ can be derived as follows:

(3) $$p\left({\left\{m_{t},m_{t}^{*}\right\}}_{t=1}^{T}|\nu ,\nu^{*}\right)=\prod_{t=0}^{T}p\left(m_{t}^{*}|m_{t},\nu -M_{t-1},\nu^{*}-M_{t-1}^{*}\right)\times p\left(m_{t}|v,\lambda \right),$$

which is nothing but the likelihood function of the total number of malignant cells that might proliferate in different regions in the brain, i.e., $L(\nu ,\nu^{*};\lambda )$.

The estimates for ν, $\nu^{*}$ and λ have been calculated using Newton–Raphson method to obtain $\hat{\nu }$, $\hat{\nu^{*}}$ and $\hat{\lambda }$; thus, we arrive at $L(\hat{\nu },\hat{\nu^{*}};\hat{\lambda })$. It must be noted that there is no special justification behind choosing the Newton–Raphson method, except that it is less time consuming compared to other numerical methods. The estimates can be obtained using any standard numerical computations.

The final part is choosing a suitable prior for our data. Suppose the number of $m_{t}^{*}$ malignant cells proliferating in the different steps follow a Poisson distribution with parameter $\lambda^{*}>1$. Then, using weighted concept, a new distribution of $m_{t}^{*}$ can be obtained as follows:

(4) $$g_{M_{t}^{\prime }}\left(m_{t}^{*}\right)=f_{M_{t}^{\prime }}\left(m_{t}^{*}\right)\times c.v.\left(m_{t}^{*}\right),$$

where $f_{M_{t}^{\prime }}\left(m_{t}^{*}\right)$ is a Poisson distribution with expectation $\left[\nu^{*}-M_{t-1}^{*}\right]\lambda^{*}$, and $c.v.\left(m_{t}^{*}\right)=\frac{1}{\sqrt{\left(\nu^{*}-M_{t-1}^{*}\right)\lambda^{*}}}<1$ is the coefficient of variation of the number of malignant cells in the *t*-th step It must be noted that $M_{t}^{\prime }$ is just a notation to indicate the distribution, and it is different from what is implied by $M_{t}$. Here, we could have just assumed $f_{M_{t}^{\prime }}$ as our prior, or we could have simply chosen the mean or standard deviation of $m_{t}^{*}$ in the product, but we wanted to make our distribution as precise as possible, particularly when it comes to prediction of no tumor cells going undetected. Thus, we go one step forward and multiply the coefficient of variation by $f_{M_{t}^{\prime }}$ to obtain our prior. This $g_{M_{t}^{\prime }}\left(m_{t}^{*}\right)$ becomes our prior distribution, i.e., the distribution of $M_{t}^{\prime }$ or the distribution for the eventual number of the malignant cells proliferating in the *t*-th step. We also estimate $\lambda^{*}$ in the above equation using Newton–Raphson method. Finally, the posterior distribution of $M_{t}^{\prime }$ can be expressed as follows:

(5) $$H_{M_{t}^{\prime }}\left(m_{t}^{*}\right)=\frac{g_{M_{t}^{\prime }}\left(m_{t}^{*}\right)\times L\left(\hat{\nu },\hat{\nu^{*}};\hat{\lambda }\right)}{\sum_{t=1}^{T}g_{M_{t}^{\prime }}\left(m_{t}^{*}\right)\times L\left(\hat{\nu },\hat{\nu^{*}};\hat{\lambda }\right)}.$$

The posterior mean is supposed to be our desired eventual number of malignant cells expected at the end of their survival time. Now, we already have information on the initial volume of the tumor for each subject when diagnosed with GBM. The eventual volume of the malignant cells may be represented as follows:

(6) $$V_{new}\propto H_{mean}\times V_{initial},$$

where $H_{mean}$ is assumed to be the posterior mean of our new distribution. A detailed explanation of the derivations of the proposed model, along with the rationale behind development of the model, have been discussed in the Appendix A, Appendix B, Appendix C and Appendix D. At the beginning when we started the testing procedure, we did not know how many voxels the tumor cells had migrated to, and so we did not know how many tests were necessary to ensure that all the tumor cells had been detected. Thus, we started predicting the eventual number at each migration step and, after a series of tests, we arrived at our final estimate. This repetition was performed with regard to each of these migration steps. The corresponding probability at each step that all malignant cells had been detected, or no malignant cells remained undetected, was determined using a realization of the geometric distribution by subjecting the number of malignant cells that were detected in each step.

### 2.4. Bayesian Regression of the Eventual Volume for Other Radiomic Features

We believe that predicting the eventual volume alone from the volume of the tumor at the time of detection is not enough, particularly when dealing with a fatal tumor such as glioblastoma. We expect the final eventual volume to depend on a number of other radiomic properties of the tumor. Thus, in this step, we must simply condition the eventual volume of the tumor obtained above on some other radiomic features of the tumor that we suspect might also contribute to the growth of the volume of the tumor. We could have just run a simple generalized linear model (GLM) to incorporate the covariates, but since we chose the response as the posterior mean obtained from (6), we deemed that a simple linear model would not truly represent the dependence or association between the response and the predictors. This is the reason we particularly made use of a Bayesian regression model. The covariates that have been included in this analysis are the different radiomic features which have been extracted volumetrically (in 3D) on the basis of manually revised labels of each of the tumor subregions. The list includes the intensity, the morphology, the histogram-based parameter, the textual parameter based on wavelets, the gray-level co-occurrence matrix (GLCM), the gray-level run-length matrix (GLRLM), the gray-level size-zone matrix (GLSZM), the neighborhood gray-tone difference matrix (NGTDM), the spatial parameter, the glioma diffusion properties extracted from glioma growth models (which have prior evaluated predictive and prognostic values), and the survival length of the tumor (measured in years). We ran a Bayesian regression model as follows:

(7a) $$y_{kl}=\alpha_{k}+\beta_{l}x_{kl}+\epsilon_{kl},k=1,\cdots ,a,l=1,\cdots ,N_{k},$$

with *a* being the number of subjects and $N_{k}$ being the number of covariates or radiomic features available for the *k*th subject. We assumed the errors were independent and identically distributed with constant variance $\sigma^{2}$ across all observations. However, this assumption does not imply that we have assumed all the predictor variables to be homogeneous among themselves. The model for the Bayesian linear regression with our conditioned response can be algebraically represented as follows:

(7b) $$Y_{kl}|x_{kl},\alpha ,\beta ∼H\left(mean=\beta^{T}X,sd=\sigma^{2}I\right),$$

where *H* is the posterior distribution obtained in the previous step, and *I* is simply the identity matrix.

Since the response was obtained from the posterior distribution above, we had to assume the model parameters α,β came from certain distributions as well. We assumed both of them to come from the Beronoulli (0.5) distribution. Then, we ran the Bayesian regression model on chosen covariates to predict the eventual volume of the tumor, and we observed if this inclusion of covariates raised the probability values of no more tumor cells remaining undetected. This probability was again calculated using the same matching technique as discussed in the previous section.

### 2.5. Handling the Heterogeneity among Oncogenes

When it comes to a prediction model with heterogeneous predictor variables, it is not a good choice to assume all variables to be homogeneous with no associations among themselves and to run a simple regression model. We know that GBM is a heterogeneous tumor; hence, it is quite likely that the oncogenes will differ from one subject to another. In other words, it is expected that the predictor variables that we wish to include to enhance our estimate of eventual volume will not share similar properties among all the subjects. Thus, in order to accommodate this heterogeneity, we modified the model in Equation (7a) and restructured it using a structural equation model (SEM) that took into account the different radiomic features of the tumor. Suppose we split these radiomic (manifest) properties into $q_{1}$ and $q_{2}$ endogenous $x_{1kl}$ and exogenous $x_{2kl}$ variables, respectively. The associations among these manifest variables can be described in terms of latent constructs using a structural equation model (SEM), as proposed by Ansari et al. (2000) [71]. Let us consider two vectors $\eta_{1kl}$ and $\eta_{2kl}$ of dimensions $d_{1}\times 1$ and $d_{2}\times 1$ for the endogenous and the exogenous latent variables, respectively. Thus, we split the covariates $x_{kl}$ in the above model and restructured them as follows:

$$x_{1kl}=\gamma_{k,x_{1}}+\Lambda_{k,x_{1}}\eta_{1kl}+\delta_{1kl}$$

(8) $$x_{2kl}=\gamma_{k,x_{2}}+\Lambda_{k,x_{2}}\eta_{2kl}+\delta_{2kl}.$$

Here, $\gamma_{k,x_{1}}$ and $\gamma_{k,x_{2}}$ are the $q_{1}\times 1$ and $q_{2}\times 1$ measurement intercept vectors for the endogenous and the exogenous variables, respectively. The $q_{1}\times d_{1}$ matrix $\Lambda_{k,x_{1}}$ and the $q_{2}\times d_{2}$ matrix $\Lambda_{k,x_{2}}$ contain the factor loadings. Furthermore, $\delta_{1kl}∼N\left(0,\Theta_{k,x_{1}}\right)$ and $\delta_{2kl}∼N\left(0,\Theta_{k,x_{2}}\right)$ represent the vectors of measurement errors with the $q_{1}\times q_{1}$ and the $q_{2}\times q_{2}$ diagonal matrices $\Theta_{k,x_{1}}$ and $\Theta_{k,x_{2}}$, respectively, which are the respective measurement error variances. This restructured model was then put to use in the form of covariates while running the Bayesian regression model as in Equation (7a).

## 3. Results

### 3.1. Simulation Study

We first conducted a simulation run to see if the proposed model yielded good results. This step was an attempt to see the degree of efficacy of the newly proposed model. In the next subsections, we have applied the model on our dataset. In our original dataset, we had 102 patients. Thus, for our simulation, we chose the same sample size and ran the simulated data.

Table 2 shows a sample of ten estimates chosen randomly. We have mentioned the case numbers or the simulation numbers under the first column. The corresponding estimates are for these respective samples. We also estimated the corresponding probability values for each case as an outcome of a geometric distribution, as discussed in the previous section. In addition, we also found the 95% confidence intervals for both the eventual volume and the corresponding probability estimates for all the cases. We found that the probability estimates were high for higher values of the posterior mean and low for lower values of the posterior mean at any given time point. This is what we had expected our model to yield—that the probability should be high enough with an increase in the posterior mean estimates. This is an indication of the fact that, as more and more tumor cells are detected—be they benign or malignant—the volume of these cells taken together increases, and, correspondingly, the probability that no tumor cells goes undetected increases. The time point, however, did not play a role in our model, and so the results were expected not to change with the change in time points. We deemed this to be a crucial point in our model, as the final predicted volume should not change with fluctuations in time points somewhere in the middle. We only considered fixed time points when we used our response later on while running a regression.

**Figure 2:**
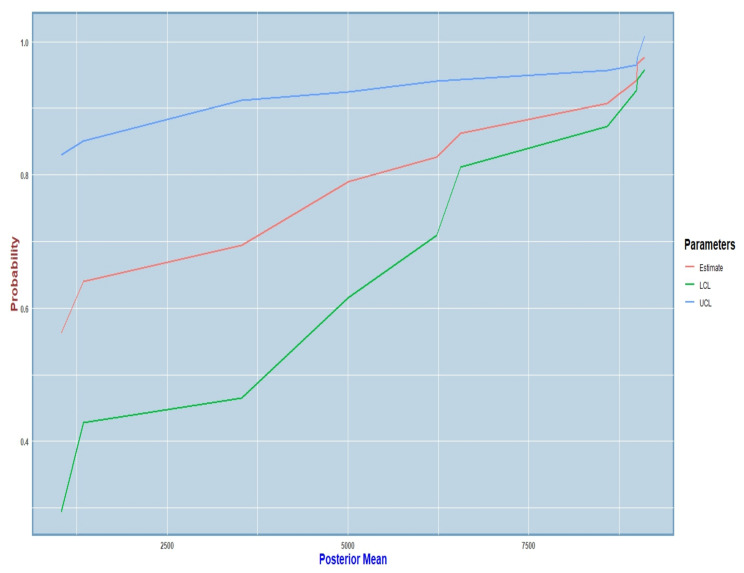
Estimated probability derived from proposed growth model and the corresponding 95% confidence intervals.

The graph in Figure 2 shows an increasing curve, which illustrates our findings more clearly. This curve was plotted for all of the 102 cases taken together. The red curve in the graph indicates our actual estimated values from the simulation. The green curve indicates the corresponding lower confidence band, and the blue curve indicates the corresponding upper confidence band at a 95% confidence level. The overall trend was increasing, thereby implying higher probability estimates for high posterior mean estimates, but the lower confidence band had values that were a bit far off from the true estimate. This happened because the standard error estimates for the probabilities were not very low in all cases, and so the values were cumulated for some points. However, we did not deem this to be a major drawback, as the desired trend was achieved, i.e., we did obtain high probability estimates with high posterior mean estimates, which we had aimed for in this case.

### 3.2. Real Data Analysis: Canonical Measurement Metrics

**Figure 3:**
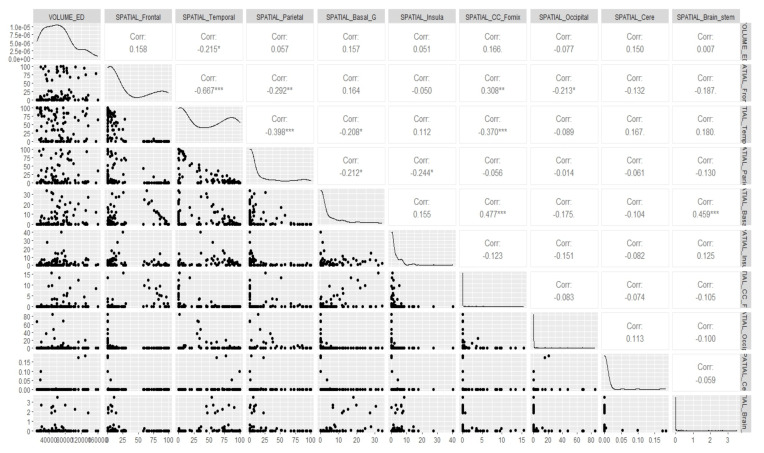
Correlation between volume and spatial features of brain for tumor region “Edema”. * *p* < 0.05, ** *p* < 0.01, *** *p* < 0.001.

**Figure 4:**
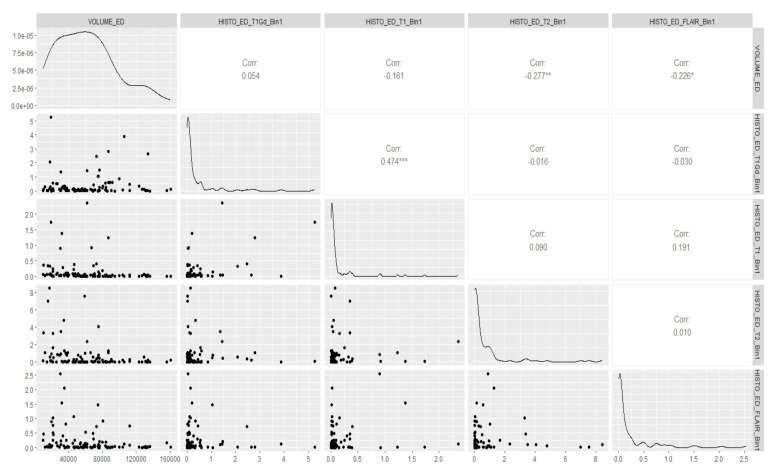
Correlation between volume and histology features of brain for tumor region “Edema”. * *p* < 0.05, ** *p* < 0.01, *** *p* < 0.001.

Before applying our model to the actual data, we started off with some fundamental canonical measurements that could help us gauge the degree of relationship between the volume of the brain tumor and its radiomic features, particularly the spatial and histology properties corresponding to the different regions of interest (ROIs) we considered. We visually examined the multivariate relationship between the volume of the tumor and its spatial features for all the five ROIs that we considered, i.e., edema (ED), GD-Enhancing tumor (ET), non-enhancing Tumor (NET), tumor core (TC) and whole tumor (WT), as well as the volume of the tumor and its histology features for the edema (ED), GD-Enhancing tumor (ET) and non-enhancing Tumor (NET), using a multivariate scatterplot. We deemed this to be a necessary step, as we wished to obtain an overall idea as to what result we should expect when we ran the model. These plots gave us an idea of the amount or relatedness among the different radiomic features of the tumors for all the subjects. Also, this measurement metrics analysis was appropriate in our case, because it was primarily a situation where we needed to execute multiple regressions, and we has multiple intercorrelated outcomes and predictor variables. The correlation plots showing the relationship between the volume of the brain and its spatial features, as well as between the volume of the brain and its histology features for the tumor subregion “Edema”, are shown in Figure 3 and Figure 4, respectively. The remaining correlation plots are included in the Appendix E.

We also made use of Wilk’s Lambda F-approximation test, which is a nonparametric test to establish the degree of linear or nonlinear relationship between the radiomic features of the brain tumor that we chose to use in our study [72,73]. The results obtained using these preliminary analyses are tabulated, as shown in Table 3.

If we look at the scatterplots, we find that there exists several significant associations between the volume and spatial features, as well as between the volume and the histology features of the tumor. Thus, it becomes clear that there is a need for considering the dependence of tumor volume on the different radiomic features regarding the prediction of its eventual growth up to the given survival time. The results in Table 3 more clearly illustrate our findings. We do not have supporting histology features for the tumor regions TC and WT, whereas we do have them for the tumor regions ED, ET, and NET. Looking at the *p*-values in the Wilk’s Lambda F-test, we can infer that there is a significant amount of association between the histology features and tumor volume for all the three ROIs considered, and there is also a significant amount of association between the spatial features and tumor volume for the ROIs NET, TC, and WT. Thus, it stands out that it was absolutely necessary to incorporate these radiomic properties in our prediction formula for the eventual tumor volume. However, let us first look at the predicted estimates upon the application of the proposed Bayesian model and the corresponding probability estimates.

### 3.3. Real Data Analysis: Prediction of the Eventual Volume of GBM

In this section, we applied the newly developed model to our dataset. The description of the dataset has already been discussed above in Section 2.1. The scale used for the measurement of the volume is cubic millimeters; hence, the figures are quite huge. Thus, for the sake of our model, we truncated the measurements to account for discrete values only. We primarily made use of the estimates of the volume of the tumor measured at different regions of interest (ROIs). The results are tabulated as shown in Table 4. Again, we have just included a sample of the patients and noted down the estimates for them. The tumor subregion for which the volumetric estimate has been obtained is also highlighted. We found that the probability of no malignant cell going undetected was adequately high when we predicted the eventual volume of the malignant cells. This held true no matter what the tumor subregion was. We have also included the 95% confidence intervals for the eventual volume estimates, as well as for the probability estimates. With the standard deviation values of each being small, we see that the confidence width happened to be significantly small. However, unlike the simulation run, the confidence width for the eventual volume was slightly larger for some cases. This might have possibly happened because we rounded up the volumetric measurements to incorporate only discrete values. Thus, the results might look a bit biased, but the relatively small confidence width for the corresponding probability estimates surpassed this biasness.

**Figure 5:**
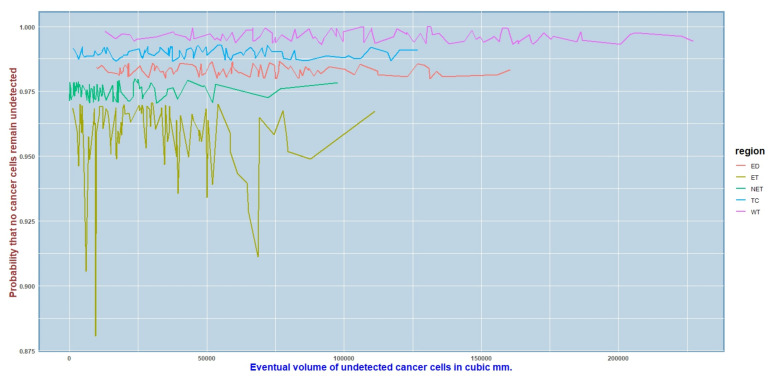
Probability that no cancer cells would remain undetected was high when we could find the eventual volume of cancer cells expected to proliferate from the tumor.

Figure 5 illustrates our findings graphically for all the subjects taken together for the different regions of interest that we incorporated in our model. The different-colored curves represent the different regions of interest (ROIs) that we considered. The yellow curve represents the tumor region “GD-Enhancing Tumor” (ET). The green and the red curves represent the tumor regions “Non-Enhancing Tumor” (NET) and the “Edema” (ED), respectively. Finally, the blue and the pink curves represent the “Tumor Core” (TC) and the “Whole Tumor” (WT), respectively. If we look at the probability values on the y-axis, we see that they were sufficiently high, starting from 0.88 and continuing up to almost 1. For the tumor region “GD-Enhancing Tumor” (ET), too many rises and falls were observed, but in no way did the probability estimates fall below 0.875. For the other tumor regions, there were not too many jumps, as opposed to the ET. The jumps in the graph, however, do not indicate that the probability estimates were decreasing with rise in the eventual volume estimate. This happened primarily because the graph was for all the subjects taken together in the order in which we had their data, and they were not in any sorted order. For some patients, if the survival time of the tumor was low, the corresponding volume of the tumor at the time of detection had been low as well; consequently, the eventual volume predicted from the model would be low as well. By “low” here, we precisely mean that the numeric figures were lower for this subject as opposed to another subject whose tumor had a higher survival time, and, hence, the higher volume of the tumor at the time of detection, the higher the predicted eventual volume estimate. However, lower eventual volume estimates did not indicate that the corresponding probability of no tumor cells remaining undetected would be low as well. In fact, this probability estimate was expected to be high for all subjects. For the ROI “Whole Tumor” (WT), the probability values remained at the maximum throughout. The high probability estimates prove the efficacy of our model.

### 3.4. Real Data Analysis: Outcome of the Bayesian Regression Model

**Figure 6:**
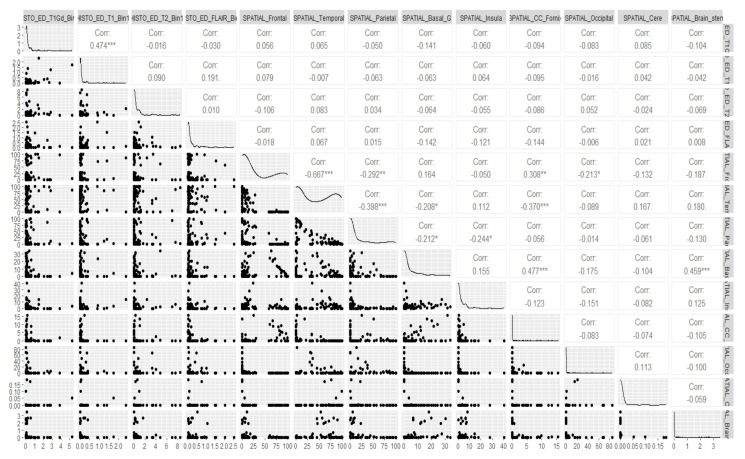
Correlation between histology and spatial features of brain for tumor region “Edema”. * *p* < 0.05, ** *p* < 0.01, *** *p* < 0.001.

In Section 3.3, we have already determined the estimates of the eventual volume of the glioblastoma expected at the end of the respective survival times of the tumors for each patient. In this section, using this eventual volume (i.e., the predicted volume from the posterior mean obtained above) as our dependent variable, we ran a Bayesian regression on all the predictor variables that we defined. The rationale behind the choice of Bayesian regression as opposed to a simple linear regression, along with the list of the covariates, have already been discussed in Section 2.4. All the radiomic features were not available for all the subjects, and we also considered the case that the manifest variables might not be the same for all the subjects. We repeated our canonical measurement metrics analysis once again and first conducted a canonical measurement among the different radiomic features that we considered to include as covariates, particularly the spatial and histology properties of the tumors. This radiomics analysis through canonical metrics enabled us to decide if other radiomic features could be heterogeneous or homogeneous. We deemed this association to be crucial as well, in addition to the inter-relatedness among the response and the predictor variables. Figure 6 shows the correlation between the spatial and histology features for the tumor region “Edema” (ED). In fact, we found it essential to account for this heterogeneity while running our regression model. The correlation plots for the ROIs “GD-Enhancing Tumor” (ET) and “Non-Enhancing Tumor” (NET) are included in the Appendix E. which also happens to show a significant amount of non-negligible correlations.

**Figure 7:**
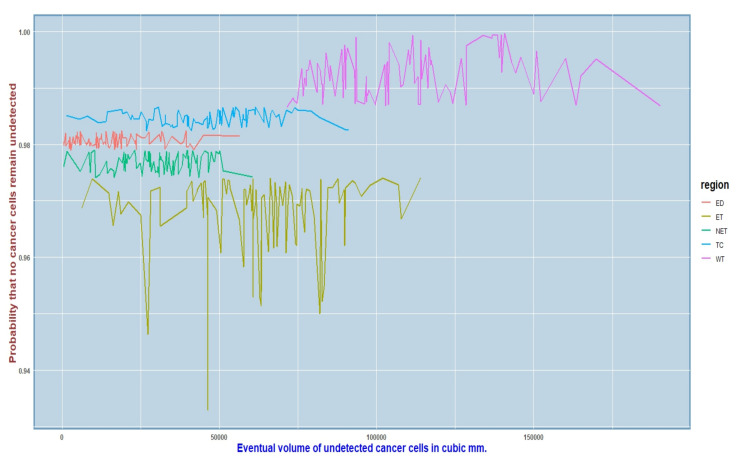
Probability that no cancer cells would remain undetected increased from the previous case when we ran a regression of the predicted eventual volume on the different radiomic features of the tumor.

We found that, although there was not much significant association among these properties for the different regions of interest that we considered, yet we could not simply reject this degree of relatedness or the case of heterogeneity, whether positive or negative. So, we deemed it fit to consider the structure equation model that accommodates heterogeneity among covariates, and we then applied the Bayesian regression model that we developed in Equation (7a). The response variable was the eventual volume of the tumor obtained from Equation (6), while the predictor variables were split into two groups—the radiomic features of the tumor as discussed in Section 2.4 comprised of the endogenous properties, while the survival length of the tumor and the age of the patient were considered to be the exogenous properties. The canonical regression quantile analysis focused on the evaluation of the performance for a much greater eventual volume of the tumor, which was subject to the summary results of the radiomic properties available up to the given survival length of the tumor for the five ROIs. We then plotted a graph for the predicted eventual volume up to the given survival time and calculated the simultaneous probability that no cancer cells had been left undetected. Applying the Bayesian regression seemed to be a good choice, as we discovered that the probability that no malignant cells were undetected increased even more as compared to the previous case. We have shown the results graphically in Figure 7 for all the subjects taken together, and we can observe that the minimum probability estimate in this case happened to be 0.94, which is extremely high and much higher as compared to the probability of 0.875 obtained earlier when only the initial volume of the tumor at the time of detection was considered. If we observe the graph carefully, the probability estimates ended up being larger for some regions of interest (ROIs) (going up to almost 1) as compared to the others, but it is noteworthy that, no matter what, the 94% chance of no malignant cells going undetected from a prediction problem is undoubtedly high. This graph includes all the subjects in our study, and the jumps occurred for the same reason that we discussed in the previous section.

Finally, we also conducted a cross-validation run of our proposed model and tabulated the results corresponding to the different tumor subregions. This analysis acted as a compare and contrast study of our results from the proposed model. The results are shown in Table 5. The comparison was performed on the basis of a simple generalized linear model (GLM). The GLM model estimates were undoubtedly good, but we have explained earlier why a GLM alone does not look good for the kind of prediction problem we are dealing with here. The R-squared estimates happened to be significantly high, except for the ROI tumor core (TC), which was, nevertheless, not very poor (greater than 50%). For the GLM case as well, we obtained a low R-squared value for the subregion TC. This could have been the result of rounding up our volume estimates during prediction, but the moderately higher estimates, higher than 80%, can be considered to be an indicator of good performance. The R-squared estimate can indeed prove how efficient the model is in predicting our response. The AIC (Akaike Information Criterion) scores were not that low, and they still can be considered as an indicator for the good performance of our model. At minimum, we found that the AIC values for our model were lower than those for the GLM model. Furthermore, we also implemented an unsupervised machine learning tool—the cross-validation using Approximate Bayesian Computation (ABC)—to evaluate the performance of our proposed model, and we discovered that the cross-validation error estimate was significantly low, being much lower than the corresponding simple generalized linear model (GLM), thereby proving the efficacy of the methodology. This tool happens to be the best one that can gauge how well a model performs through its evaluation of the error in the prediction. Since we have the volume estimates in cubic millimeters, and our figures are immensely huge, this relatively small amount of error might be admissible.

## 4. Discussions

The main attempt in this work was to come up with a suitable radiomics model that could fasten the prediction of the growth of a glioblastoma multiforme (GBM) once it was detected. It is known that once a tumor is born, it can grow only upto a certain threshold or survival time. Once it reaches this threshold, it dies or is removed by surgery. The target was to predict this threshold volume on the basis of the number of tumor cells that might proliferate in the entire brain from this tumor. Another crucial factor in our case while dealing with a fourth-grade malignant tumor was that we did not have a scope for the nonsurgical treatment of the tumor, and so the death of the tumor only implied its surgical extraction. In other words, the survival time of the tumor is actually the maximum possible threshold time of the growth of the tumor, after which the patient will die if the tumor is still not removed. For implementing our prediction model, a simple approach was used based on the Classical Occupancy Problem in Probability Theory [74], which was also extended to a Bayesian framework. The initial determination of the exact location of the tumor and the masking of its image using parallel image segmentation seemed to be a wise choice for the application of our model, as we knew beforehand the precise location of the tumor. Determining this exact location enabled us to account for the assumption of newborn and migratory tumor cells in our model, i.e., we could more reliably keep track of the new tumor cells being born from the original tumor and could also identify the cells that had migrated from some other location. In addition, the use of the image intensities and the eventual identification of the prognostic markers are an improvement upon previous research conducted on multiple lesion glioblastoma (M-GBM), where the multiple foci of tumor enhancement were found to result in inferior prognoses of single-lesion GBM cases [75,76]. The image segmentation algorithm we used is slightly a better version when compared to the multidimensional analysis performed by Armocida et al. (2021), which showed a higher proportion of tumors with EGFRvIII mutations in M-GBM cases, but could not verify whether there was a genetic or a molecular multi = focality basis [75]. Our model seems to be an efficient one in any given prediction problem, as we discovered that the probability of no cancer cells remaining undetected once the eventual volume of the tumor was obtained happened to be greater than 87%, which is definitely very high. Another interesting point to be noted is that this probability further increased to 94% when we considered the different clinical and demographic characteristics of the patients, along with the different radiomic parameters of their tumors. This happens to be at par with our findings from canonical measurements that showed a significant dependence of the tumor volume on different radiomic features. In our analysis using image segmentation, we successfully overcame the gaps pertaining to clinician input to identify the relevant regions of interest (ROIs). Although medical research claims clinician information to be the gold standard, deep-learning strategies or radiomics statistical models such as ours have the potential to define ROIs without the bias of human segmentation [77]. In our simulated data, we found that the confidence widths for the eventual volume, as well as for the corresponding probabilities, were relatively small. While for the actual data, there was a bit deviation in the fact that the confidence width of the eventual volume happened to be a bit high, but that for the probability estimates was relatively small.

However, the model has certain drawbacks as well. First of all, image segmentation only gives the location of the tumor when detected. We assumed (and it is definite to assume) that there would be migratory cancer cells as well, but the locations of other malignant cells, whether migratory or new, are unknown. We assumed different voxels in the brain where the tumor cells could have migrated, but we are not aware of the exact positions of the voxels. In addition, the scale used for the measurement of volume was cubic millimeters, and, hence, the figures are quite large. Thus, for the sake of convenience, we rounded up the figures and only considered discrete values to incorporate the data in our model. This might have resulted in a bit of bias in our model. The model is expected to perform efficiently only when we have discrete values in a dataset. Of course, predicting a suitable model for continuous outcomes or a similar model incorporating varying time points can be a scope of future work. This can be probably made possible by extending the Bayesian framework in the form of a partial differential equation to account for the change in time points, which was challenging here. This was particularly not possible for this study, because we only had a fixed survival time of the tumors and no further information. However, a positive contribution of the model is that it is not subject to changes in time; thus, the final predicted volume is more reliable, as there is no scope for different predictions when time points change.

## 5. Conclusions

It is true that glioblastoma remains a challenging disease to treat, and current standard treatments are often limited in their efficacy [78]. However, there have been some promising developments in the field, including the use of animal models to better understand the disease and to test potential treatments. One promising approach is the use of targeted therapies that focus on specific molecular pathways that are involved in the development and growth of glioblastoma. These therapies aim to exploit the genetic and molecular abnormalities that are characteristic of the disease, and many are currently being tested in clinical trials [79,80,81]. Again, stochastic variational inference, which is a specific technique within Bayesian modeling, is often used to approximate the posterior distribution of the model parameters. This is necessary, because in many cases, the posterior distribution cannot be calculated analytically; instead, an iterative algorithm must be used to approximate the posterior distribution by minimizing a divergence measure between the approximate distribution and the true posterior. This allows us to make predictions and draw inferences based on the model [82]. Overall, Bayesian modeling and stochastic variational inference are powerful tools in the case of data similar to ours, which can help in predicting the growth of fatal tumors such as glioblastoma and other complex phenomena [83]. By using these techniques, we can gain a better understanding of the underlying dynamics and make more informed decisions about patient care [84,85,86]. In addition, there is a growing interest in using immunotherapy to treat glioblastoma, which works by activating the patient’s immune system to recognize and attack the cancer cells. Although this approach is still in the early stages of development, early studies have shown some promise in using immunotherapy to treat glioblastoma [87,88,89,90]. Overall, it is clear that there is a need for continued research and the development of new treatments for deadly tumors such as GBM. With the use of animal models and other advanced technologies, scientists and clinicians are making progress toward a better understanding of the disease and toward developing more effective therapies to treat it [88,89,91,92,93,94,95,96]. It might be worthwhile to amalgamate all such information into an artificial intelligence (AI) radiomics model that can perhaps be a promising direction to effecting advance personalized medicine, and such as model may direct this field towards the upgradation of radiomics in the early diagnosis of glioblastoma [97].

## Figures and Tables

**Table 1:** Summary statistics of different parameters and radiomic features of GBM tumors.

Radiomic Features	Parameters	Mean	Standard Deviation
Volume	Whole Tumor	107,999.84	52,700.74
	Edema	62,139.61	35,360.39
	Tumor Core	45,560.24	31,424.16
	Non-enhancing Tumor	15,578.29	17,475.42
	GD-Enhancing Tumor	29,981.94	22,104.20
Spatial Parameters	Spatial Frontal	25.64	35.69
	Spatial Temporal	42.39	38.89
	Spatial Occipital	4.22	14.13
	Spatial Insula	2.95	5.76
	Spatial Fornix	1.19	3.04
	Spatial Parietal	18.34	29.50
	Spatial Brain Stem	0.25	0.71
Histology Parameters	Occipital Cortex	0.375	0.8097
	Temporal Cortex	0.146	0.3546
	Basal Ganglia	0.681	1.508
Morphology	Eccentricity	0.68	0.09
	Solidity	0.40	0.14
Survival Length (in years)		1.5	1.4

**Table 2:** Posterior mean values and the corresponding probabilities up to a given fixed time point obtained from the simulated data. *Note: The time point did not play any role in this simulation*.

Sample No.	Posterior Mean Volume		Probability	
	**Estimate**	**95% C.I.**	**Estimate**	**95% C.I.**
43	1038	[1037.924, 1038.076]	0.5625000	[0.2944, 0.8306]
51	1346	[1345.911, 1346.089]	0.6400000	[0.4286, 0.8514]
8	3528	[3527.910, 3528.090]	0.6944444	[0.4643, 0.9125]
65	5008	[5007.907, 5008.093]	0.7901235	[0.6155, 0.9245]
101	6224	[6223.896, 6224.104]	0.8264463	[0.7092, 0.9408]
96	6559	[6558.842, 6559.158]	0.8622449	[0.8119, 0.9436]
12	8587	[8586.837, 8587.163]	0.9070295	[0.8732, 0.9570]
38	8990	[8989.836, 8990.164]	0.9420415	[0.9271, 0.9647]
17	9001	[9000.813, 9001.187]	0.9674819	[0.9430, 0.9771]
74	9101	[9100.723, 9101.277]	0.9760488	[0.9579, 0.9990]

**Table 3:** Significance of radiomics features based on Wilk’s Lambda F-approximation test.

ROIs		Spatial Features			Histology Features	
	**F-Stat.**	***p*** **-Val.**	$\rho_{\mathrm{vol},\mathrm{spatial}}$	**F-Stat.**	***p*** **-Val.**	$\rho_{\mathrm{vol},\mathrm{histology}}$
ED	0.8814	0.2104	0.3445	0.8540	0.0381	0.3826
ET	0.8601	0.1083	0.3746	0.8676	0.0076	0.3638
NET	0.7899	0.0074	0.4584	0.8822	0.0154	0.3432
TC	0.7805	0.0012	0.4685	-	-	-
WT	0.7820	0.0052	0.4669	-	-	-

**Table 4:** Eventual volume of cancer cells (predicted based on the model) and the corresponding probabilities that no cancer cell would remain undetected up to the survival time of the tumor obtained from the actual data for a sample of subjects.

Eventual Volume (In Nearest Cubic mm)		Probability That No Cancer Cells Remain Undetected		Tumor Subregion
**Estimate**	**95% C.I.**	**Estimate**	**95% C.I.**	
9525	[9433.78, 9616.22]	0.9937805	[0.9934967, 0.9940643]	ET
68,592	[68,500.78, 68,683.33]	0.9961458	[0.9958620, 0.9964296]	ET
5899	[5807.78, 5990.22]	0.9556447	[0.9553609, 0.9559285]	ET
31,614	[31,522.78, 31,705.22]	0.9907852	[0.9905014, 0.9910690]	NET
7338	[7246.78, 7429.22]	0.8806554	[0.8803716, 0.8809392]	NET
17,679	[17,587.78, 17,770.22]	0.9778719	[0.9775881, 0.9781557]	NET
34,935	[34,843.78, 35,026.22]	0.9738203	[0.9735365, 0.9741041]	ED
70,998	[70,906.78, 71,089.22]	0.9777224	[0.9774386, 0.9780062]	ED
83,517	[83,425.78, 83,608.22]	0.9890068	[0.9887230, 0.9892906]	ED
117,105	[117,013.78, 117,196.22]	0.9839206	[0.9836368, 0.9842044]	TC
86,271	[86,179.78, 86,362.22]	0.9632160	[0.9629322, 0.9634998]	TC
37,513	[37,421.78, 37,604.22]	0.9502363	[0.9499525, 0.9505201]	TC
279,108	[278,136.78, 281,916.22]	0.9618206	[0.9536135, 0.9871034]	WT
196,472	[183,412.78, 203,120.22]	0.9931245	[0.9910266, 0.9954483]	WT
138,532	[113,574.78, 158,964.22]	0.9802536	[0.9795436, 0.9871256]	WT

**Table 5:** Cross-validation results obtained using R-squared, Akaike Information Criterion (AIC), and an unsupervised learning tool. *Note: “Cross Validation” has been abbreviated here as C.V*.

ROIs	R-Squared		AIC		C.V. Error	
	**GLM**	**Bayesian**	**GLM**	**Bayesian**	**GLM**	**Bayesian**
ED	0.7936	0.8341	212.56	179.69	0.3637	0.2931
ET	0.8924	0.9018	265.23	259.86	0.7750	0.7236
NET	0.8825	0.9341	338.53	338.38	2.1869	1.1805
TC	0.5301	0.5305	301.25	251.61	0.7304	0.7293
WT	0.9176	0.9372	190.46	187.35	0.3634	0.3130

**Figure A1:**
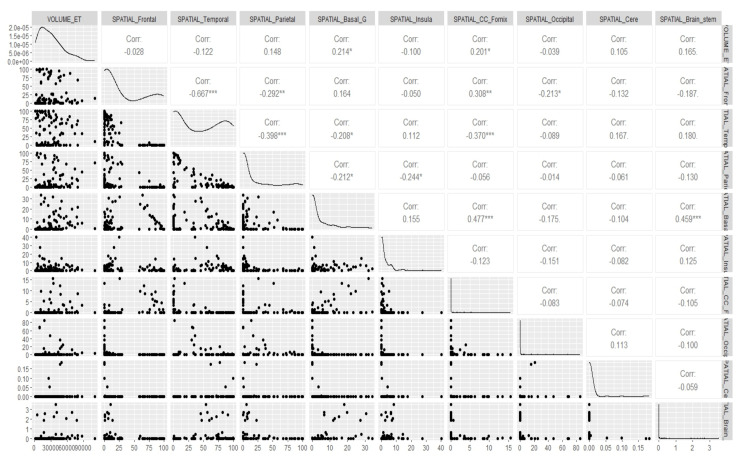
Correlation between volume and spatial features of brain for the region ET. * *p* < 0.05, ** *p* < 0.01, *** *p* < 0.001.

**Figure A2:**
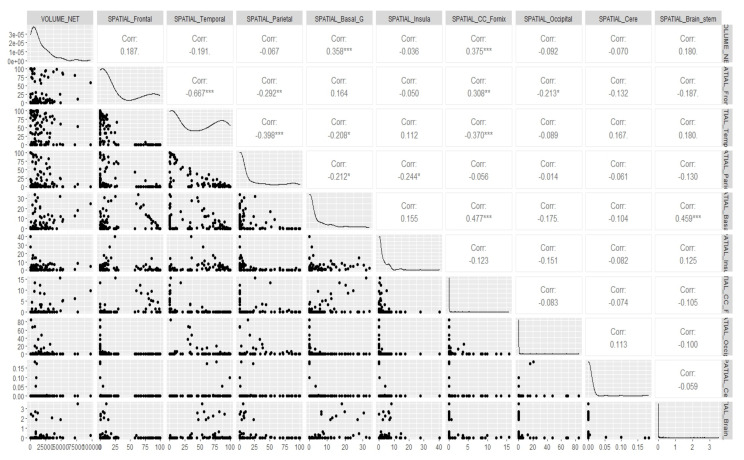
Correlation between volume and spatial features of brain for the region NET. * *p* < 0.05, ** *p* < 0.01, *** *p* < 0.001.

**Figure A3:**
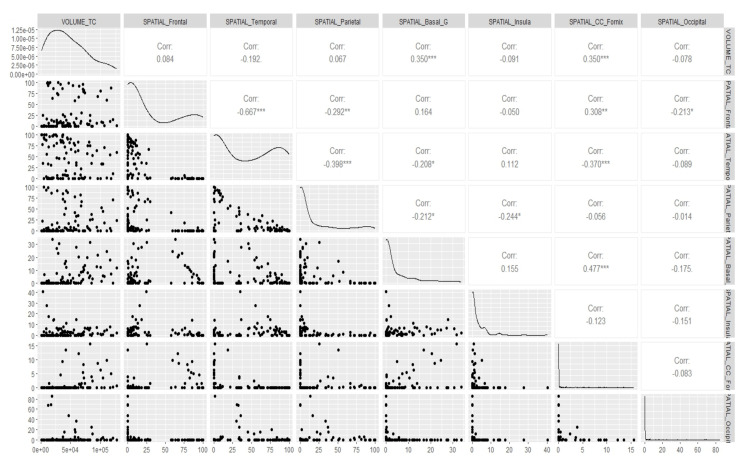
Correlation between volume and spatial features of brain for the region TC. * *p* < 0.05, ** *p* < 0.01, *** *p* < 0.001.

**Figure A4:**
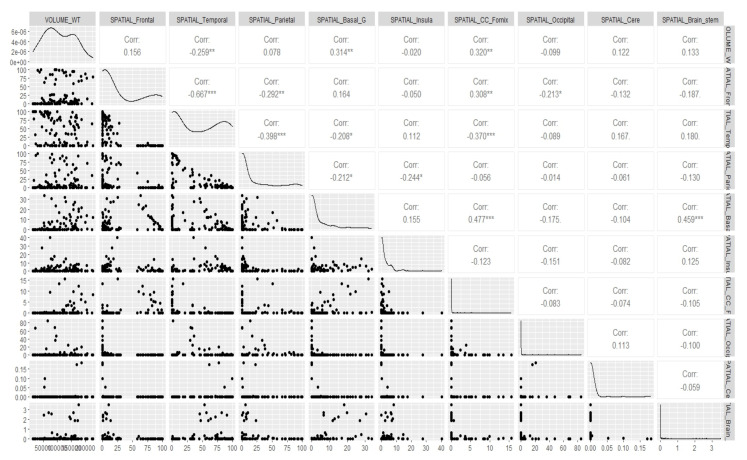
Correlation between volume and spatial features of brain for the region WT. * *p* < 0.05, ** *p* < 0.01, *** *p* < 0.001.

**Figure A5:**
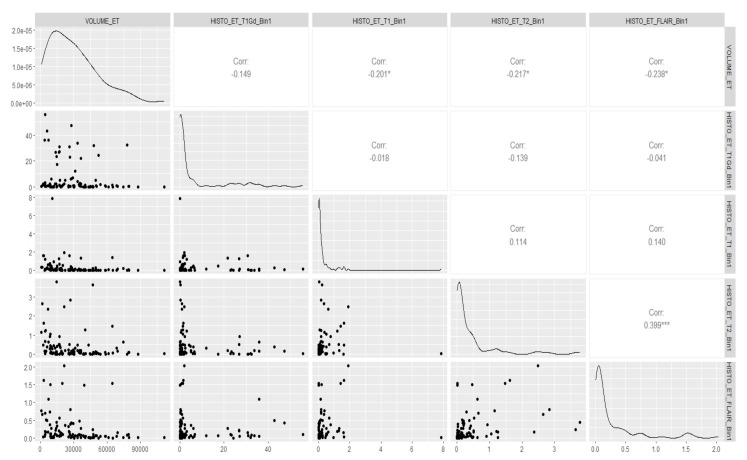
Correlation between volume and histology features of brain for the region ET. * *p* < 0.05, *** *p* < 0.001.

**Figure A6:**
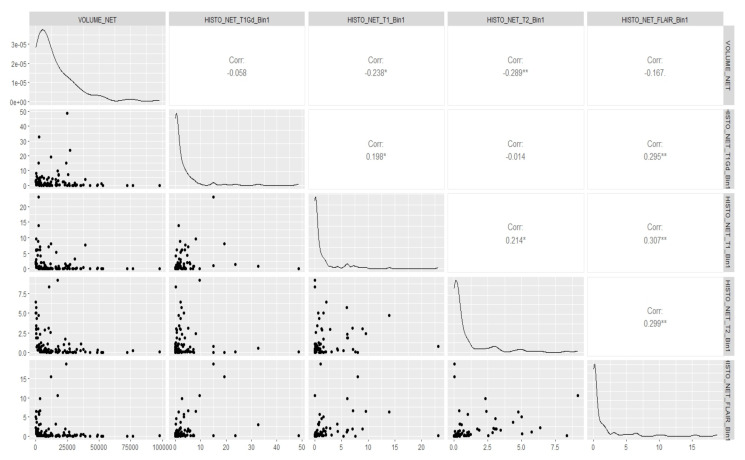
Correlation between volume and histology features of brain for the region NET. * *p* < 0.05, ** *p* < 0.01.

**Figure A7:**
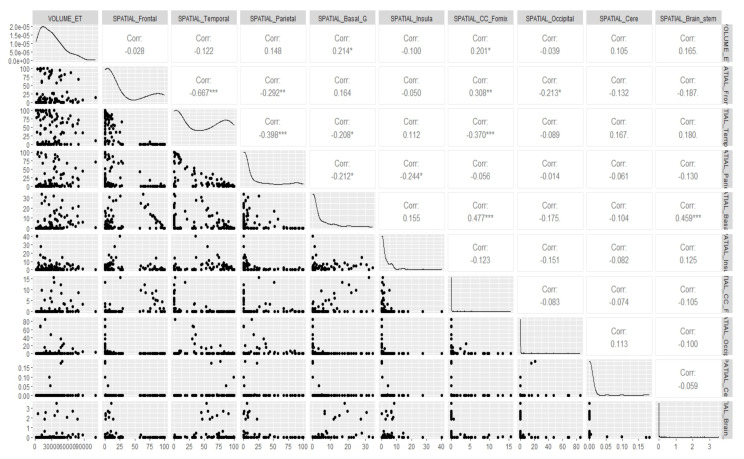
Correlation between histology and spatial features of brain for the region ET. * *p* < 0.05, ** *p* < 0.01, *** *p* < 0.001.

**Figure A8:**
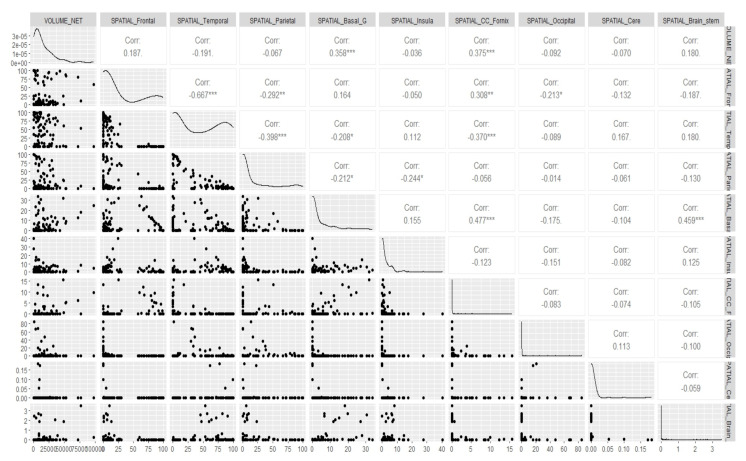
Correlation between histology and spatial features of brain for the region NET. * *p* < 0.05, ** *p* < 0.01, *** *p* < 0.001.

## Data Availability

The data presented in this study are available in this article.

## Abbreviations

The following abbreviations are used in this manuscript:

GBM	Glioblastoma Multiforme
ROIs	Regions of Interest
ET	GD-Enhancing Tumor
TC	Tumor core
WT	Whole Tumor
ED	Edema
NET	Non-Enhancing Tumor

## Appendix A. Background behind Deriving the Model

Let us consider a distribution for the number of $m_{t}^{*}$ malignant cells proliferating in the *t*-th migration step. This distribution will depend on the overall volume of the tumor cells that are actually expected to proliferate from the tumor, and our target is to find that out. The main motive behind developing this distribution is that, if this is determined beforehand, medical procedures can be initiated to control the growth of these cells. Here, $M_{t}^{*}=\sum_{j=1}^{t}m_{j}^{*}$, i.e., the total number of malignant cells distinctly proliferated till the *t*-th migration step and $M_{t}=\sum_{j=1}^{t}m_{j}$, i.e., the total number of cells (malignant and benign) that is distinctly proliferated until the *t*-th migration step. In addition, $M_{t}$ and $M_{t}^{\prime }$ are different, as was discussed in the main paper.

## Appendix B. The Probability Model

Assume that the tumor cells have been detected in the first (t−1) migration steps; start this process in the *t*-th step after the detection procedures have been completed up to the (t−1)-th step. We first consider the volume of $\nu^{*}-M_{t}^{*}$ malignant cells that remain undetected after the patient is diagnosed with GBM. Let $A_{x}$ be the event that the malignant cell *x* is detected, where $x=1,\cdots ,\nu^{*}-M_{t-1}^{*}$. Here, we assume *x* to take values up to the (t−1)-th step, because we want to ensure that the final step is free from any tumor cell proliferation, i.e., all the cells expected to proliferate have been detected once we complete testing up to (t−1) steps. Therefore, the possible number of undetected malignant cells in the *t*-th step given one malignant cell will have been detected to be ${\left(\nu^{*}-M_{t-1}^{*}-1\right)}^{m_{t}^{*}-1}$. Similarly, when two malignant cells, say *x* and *y*, are detected, there will be ${\left(\nu^{*}-M_{t-1}^{*}-2\right)}^{m_{t}^{*}-2}$ possible undetected malignant cells, and so on. In general, the probability of each of these events will be

$$p_{x}=\frac{{\left(\nu^{*}-M_{t-1}^{*}-1\right)}^{m_{t}^{*}-1}}{{\left(\nu^{*}-M_{t-1}^{*}\right)}^{m_{t}^{*}}}$$

$$p_{xy}=\frac{{\left(\nu^{*}-M_{t-1}^{*}-2\right)}^{m_{t}^{*}-2}}{{\left(\nu^{*}-M_{t-1}^{*}\right)}^{m_{t}^{*}}}$$

$$p_{xyz}=\frac{{\left(\nu^{*}-M_{t-1}^{*}-3\right)}^{m_{t}^{*}-3}}{{\left(\nu^{*}-M_{t-1}^{*}\right)}^{m_{t}^{*}}}$$

⋯

⋯

$$p_{xyz\cdots^{\;}r}=\frac{{\left(\nu^{*}-M_{t-1}^{*}-r\right)}^{m_{t}^{*}-r}}{{\left(\nu^{*}-M_{t-1}^{*}\right)}^{m_{t}^{*}}}.$$

If we go on adding these probabilities with an equal number of subscripts, we have $S_{1}=\sum p_{x},S_{2}=\sum p_{xy},S_{3}=p_{xyz}$, and, in this manner, for every discrete $r\leq \nu^{*}-M_{t-1}^{*}$, we have

Sr=ν*−Mt−1*r(ν*−Mt−1*−r)mt*−r(ν*−Mt−1*)mt*.

**Lemma** **A1**.
*We know from the fundamental rules of probability that, if $A_{1}$ and $A_{2}$ are two events, then $A_{1}\cup A_{2}$ is the event that either $A_{1}$ or $A_{2}$ or both occur. The probability of this event will be $P\left(A_{1}\cup A_{2}\right)=P\left(A_{1}\right)+P\left(A_{2}\right)-P\left(A_{1}\cap A_{2}\right)$. Similarly, for n events, we have the event $A_{1}\cup A_{2}.\ldots \cup A_{n}$. If we want to find the probability of this event, we make use of $p_{i}=P\left(A_{i}\right),$$p_{ij}=P\left(A_{i}A_{j}\right),p_{ijk}=P\left(A_{i}A_{j}A_{k}\right)$, and so on. Therefore, the probability of the realization of two events may be given by $S_{1}-S_{2}$. In general, for U events, we have $S_{1}-S_{2}+S_{3}-S_{4}+\ldots +{\left(-1\right)}^{U-1}S_{U}$.*

In our problem, we have $x<y<z<\ldots <\nu^{*}-M_{t-1}^{*}$ such that in the sums, each combination, appear once and only once. Thus, $S_{r}$ has ν*−Mt−1*r terms. Thus, the probability that $\left(\nu^{*}-M_{t-1}^{*}\right)$ cells remain undetected will be

$$S_{1}-S_{2}+S_{3}-S_{4}+\ldots +{\left(-1\right)}^{\nu^{*}-M_{t-1}^{*}-1}S_{\nu^{*}-M_{t-1}^{*}},$$

and, hence, the probability that at least one undetected malignant cell gets detected will be its complementary, or

$$1-S_{1}+S_{2}-S_{3}+S_{4}-.\ldots +{\left(-1\right)}^{\nu^{*}-M_{t-1}^{*}}S_{\nu^{*}-M_{t-1}^{*}},$$

and we denote this as $p_{0}\left(m_{t}^{*},\nu^{*}-M_{t-1}^{*}\right)$, i.e.,

(A1) p0(mt*,ν*−Mt−1*)=∑r=0ν*−Mt−1*(−1)rν*−Mt−1*r(ν*−Mt−1*−r)mt*−r(ν*−Mt−1*)mt*.

The next step is to find out the probability that all these $\left(\nu^{*}-M_{t}^{*}\right)$ undetected malignant cells will be detected. The testing is completed up to the (t−1)th migration step, and so these undetected cells can occur in ν*−Mt−1*mt* ways. Similarly, after testing up to the (t−1)th step, the malignant cells in the *t*-th step can occur in ν*−Mt−1*mt* ways. Thus, we can assume that the probability that all undetected malignant cells are detected can be a realization of the hypergeometric distribution, and we have

(A2) pν*−Mt*(mt*∣mt,ν−Mt−1,ν*−Mt*)=ν*−Mt−1*mt*ν−ν*−Mt−1+Mt−1*mt−mt*ν−Mt−1mt.

Now, if $p_{\nu^{*}-M_{t}^{*}}\left(m_{t}^{*}|m_{t},\nu -M_{t-1},\nu^{*}-M_{t-1}^{*}\right)\to 0$, it means that the malignant cells have not been detected, but there some still might occur to detect. However, if $p_{\nu^{*}-M_{t}^{*}}\left(m_{t}^{*}|m_{t},\nu -M_{t-1},\nu^{*}-M_{t-1}^{*}\right)\to 1$, as expected in this case, we can expect that all the undetected malignant cells have been detected.

## Appendix C. Finding the Likelihood Function

Here, we attempt to find the likelihood function of the total number of malignant cells that might proliferate in different regions in the brain. Let this be $L(\nu ,\nu^{*};\lambda )$. When we are calculating this likelihood, we should remember that the malignant cells are actually a subset of all the tumor cells. Thus,

$$L\left(\nu ,\nu^{*};\lambda \right)=\prod_{t=1}^{T}P\left[m_{t}^{*},m_{t}\right]=\prod_{t=1}^{T}P\left[m_{t}^{*}|m_{t},\nu -M_{t-1},\nu^{*}-M_{t-1}^{*}\right]\times P\left[m_{t}\right].$$

Since we have made an assumption that no recovery takes place till one cell has proliferated in a given stage, no matter whether it is malignant or benign, therefore, we can take the occurrence of tumor cells in the first step to be Poisson with a rate νλ. Thus, the distribution of $m_{1}$ can be taken to be Poisson with the expectation νλ.

In general, we can take the occurrence of tumor cells in the *t*-th step to be Poisson with a rate νλ, and, thus, the distribution of $m_{t}$ should be Poisson with the expectation νλ. Now, we are concentrating on the *t*-th step when the testing in (t−1) migration steps are over. Thus, the remaining cells that will occur in this step must be among $\nu -M_{t-1}$ cells. Therefore,

$$P\left[m_{t}\right]=exp\left(-\left(\nu -M_{t-1}\right)\lambda \right)\frac{{\left[\left(\nu -M_{t-1}\right)\lambda \right]}^{m_{t}}}{m_{t}!}.$$

Therefore, we have

(A3) L(ν,ν*;λ)=∏t=1TP[mt*∣mt,ν−Mt−1,ν*−Mt−1*]×P[mt]=∏t=1Tν*−Mt−1*mt*ν−ν*−Mt−1+Mt−1*mt−mt*ν−Mt−1mt×exp(−(ν−Mt−1)λ)[(ν−Mt−1)λ]mtmt!,

and, by using the Newton–Raphson method to estimate $\hat{\nu }$, $\hat{\nu^{*}}$ and $\hat{\lambda }$, we arrive at $L(\hat{\nu },\hat{\nu^{*}},\hat{\lambda })$.

## Appendix D. Choice of Prior and Posterior

Suppose the number of $m_{t}^{*}$ malignant cells proliferating in different brain voxels follows a Poisson distribution with the parameter $\lambda^{*}>1$. Then, by using the weighted concept, we propose a distribution of $m_{t}^{*}$ as

$$g_{M_{t}^{\prime }}\left(m_{t}^{*}\right)=f_{M_{t}^{\prime }}\left(m_{t}^{*}\right)\times c.v.\left(m_{t}^{*}\right).$$

Again, we concentrate on the *t*-th step when testing is complete for the (t−1) migration steps, and so the Poisson distribution in this case is expected to have a parameter $\left[\nu^{*}-M_{t-1}^{*}\right]\lambda^{*}$. As a result,

$$f_{M_{t}^{\prime }}\left(m_{t}^{*}\right)=exp\left(-\left(\nu^{*}-M_{t-1}^{*}\right)\lambda^{*}\right)\frac{{\left[\left(\nu^{*}-M_{t-1}^{*}\right)\lambda^{*}\right]}^{m_{t}^{*}}}{m_{t}^{*}!},$$

and $c.v.\left(m_{t}^{*}\right)=\frac{1}{\sqrt{\left[\nu^{*}-M_{t-1}^{*}\right]\lambda^{*}}}<1$ is the coefficient of variation of the number of malignant cells in the *t*-th migration step. As $\lambda^{*}$ increases, $c.v(m_{t}^{*})$ becomes smaller and smaller. Therefore, $\sum g_{M_{t}^{\prime }}\left(m_{t}^{*}\right)\to 1$. Since, we can see that this sum tends to 1, $g_{M_{t}^{\prime }}\left(m_{t}^{*}\right)$ becomes our prior distribution of $M_{t}^{\prime }$, i.e., the eventual number of the malignant cells proliferating in the *t*-th migration step. $\lambda^{*}$ is estimated using the Newton–Raphson method. Now, we have seen that $p_{\nu^{*}-M_{t}^{*}}\left(m_{t}^{*}|m_{t},\nu -M_{t-1},\nu^{*}-M_{t-1}^{*}\right)\to 1$ for a large malignant cell count. Furthermore, since we have assumed $P[m_{t}]$ to follow a Poisson distribution, it tends to 1. Thus, our estimated likelihood value $L(\hat{\nu },\hat{\nu^{*}};\hat{\lambda })\to 1$. Furthermore, we arrive at the posterior distribution of $M_{t}^{\prime }$ as

(A4) $$H_{M_{t}^{\prime }}\left(m_{t}^{*}\right)=\frac{g_{M_{t}^{\prime }}\left(m_{t}^{*}\right)\times L\left(\hat{\nu },\hat{\nu^{*}};\hat{\lambda }\right)}{\sum_{t=1}^{T}\left(m_{t}^{*}\right)\times L\left(\hat{\nu },\hat{\nu^{*}};\hat{\lambda }\right)}.$$
